# Shortening of the *Lactobacillus paracasei* subsp. *paracasei* BGNJ1-64 AggLb Protein Switches Its Activity from Auto-aggregation to Biofilm Formation

**DOI:** 10.3389/fmicb.2016.01422

**Published:** 2016-09-08

**Authors:** Marija Miljkovic, Iris Bertani, Djordje Fira, Branko Jovcic, Katarina Novovic, Vittorio Venturi, Milan Kojic

**Affiliations:** ^1^Laboratory for Molecular Microbiology, Institute of Molecular Genetics and Genetic Engineering, University of BelgradeBelgrade, Serbia; ^2^Bacteriology Group, International Centre for Genetic Engineering and Biotechnology, Area Science ParkTrieste, Italy; ^3^Department of Biochemistry, Faculty of Biology, University of BelgradeBelgrade, Serbia

**Keywords:** AggLb, collagen binding domains, CnaB-like domains, auto-aggregation, biofilm formation

## Abstract

AggLb is the largest (318.6 kDa) aggregation-promoting protein of *Lactobacillus paracasei* subsp. *paracasei* BGNJ1-64 responsible for forming large cell aggregates, which causes auto-aggregation, collagen binding and pathogen exclusion *in vitro.* It contains an N-terminus leader peptide, followed by six successive collagen binding domains, 20 successive repeats (CnaB-like domains) and an LPXTG sorting signal at the C-terminus for cell wall anchoring. Experimental information about the roles of the domains of AggLb is currently unknown. To define the domain that confers cell aggregation and the key domains for interactions of specific affinity between AggLb and components of the extracellular matrix, we constructed a series of variants of the *aggLb* gene and expressed them in *Lactococcus lactis* subsp. *lactis* BGKP1-20 using a lactococcal promoter. All of the variants contained a leader peptide, an inter collagen binding-CnaB domain region (used to raise an anti-AggLb antibody), an anchor domain and a different number of collagen binding and CnaB-like domains. The role of the collagen binding repeats of the N-terminus in auto-aggregation and binding to collagen and fibronectin was confirmed. Deletion of the collagen binding repeats II, III, and IV resulted in a loss of the strong auto-aggregation, collagen and fibronectin binding abilities whereas the biofilm formation capability was increased. The strong auto-aggregation, collagen and fibronectin binding abilities of AggLb were negatively correlated to biofilm formation.

## Introduction

*Lactobacillus* strains could exhibit probiotic characteristics, which confer a variety of beneficial health effects on the host and they have a number of features that make it particularly suitable for dairy applications (Salminen et al., 1998; Lebeer et al., 2008; Sisto and Lavermicocca, 2012; Giraffa, 2014). *Lactobacillus* effector molecules that contribute to the health-promoting interactions with the host (intestinal) system are likely located in the bacterial cell envelope (Bron et al., 2004; Kleerebezem et al., 2010; Hymes et al., 2016). It was found that adhesion of lactobacilli to components of the extracellular matrix (ECM) such as mucin, fibronectin, collagen, laminin, or fibrinogen may thus have a direct impact on their probiotic function, e.g., in preventing the adhesion to and the colonization of damaged intestinal tissue sites by invading pathogens (Lorca et al., 2002). It has been reported that damage of the mucosal layer of the ECM can result in its colonization by pathogens, resulting in subsequent infection (Styriak et al., 2003).

The ability of pathogenic bacteria to adhere to distinct components of the ECM, such as collagen and fibronectin, is enabled or facilitated by the expression of ECM-binding proteins, termed adhesins. Adhesins are important virulence factors of pathogens, as they are involved in the initiation of infection (Flock, 1999). Group A streptococci (GAS, *Streptococcus pyogenes*) have evolved a number of surface-bound and secreted virulence factors, of which the M proteins are probably the best characterized. Binding of GAS to epithelial cells involves an interaction between M protein and fibronectin (Oehmcke et al., 2010). Epithelial cell invasion by Group B *Streptococcus* (GBS) is associated with expression of alpha C protein (Bolduc and Madoff, 2007). Aggregation protein encoded by *asp1* gene of enterococci, characterized as a virulence factor of 142 kDa plays a crucial role in adherence to eukaryotic cells (Galli et al., 1990). In the skin abscess model, a sortase-deficient *Staphylococcus aureus* strain lacking all of its cell-wall anchored proteins was less virulent than its wild-type strain. Also, strains specifically lacking protein A, fibronectin binding proteins, clumping factor A or surface protein SasF were impaired in their virulence (Josefsson et al., 2008; Kwiecinski et al., 2014). In addition some biofilm factors related to aggregation ability, for example, Bap protein of *S. aureus* facilitates the persistence in the mammary gland by enhancing adhesion to epithelial cells and prevents cellular internalization through the binding to GP96 host receptor (Taglialegna et al., 2016).

Since systematic analysis of efficacy of probiotic therapy demonstrated that probiotic activities are strain-specific (Hungin et al., 2013; Sanders et al., 2013) the paradigm of probiotic research is rightfully shifting toward understanding the mechanistic action of each specific strain (Johnson and Klaenhammer, 2014). It has been demonstrated that the purified collagen binding protein (Cbp) from *L. plantarum* 91 possess anti-adhesion activity against the enteric pathogen *Escherichia coli* 0157:H7 on immobilized collagen (Yadava et al., 2013). Surface fibronectin binding protein from *L. casei* BL23 participates in cell attachment to immobilized fibronectin (Muñoz-Provencio et al., 2010). Also, binding of immobilized collagen and fibronectin by *L. acidophilus* CRL 639 depends on cell-surface proteins (Lorca et al., 2002). The S-layer proteins of *L. crispatus* ZJ001 also inhibited the adhesion of *Salmonella typhimurium* and *E. coli* O157:H7 to HeLa cells (Chen et al., 2007). In addition, the S-layer protein associated with moonlighting proteins acted as an adherence factor, which has been evidenced by the high capability of adhesion, auto- and co-aggregation of *L. helveticus* T159 (Waśko et al., 2014).

The ability of lactobacilli to form multicellular aggregates is an important property for colonization of the oral cavity, human gut or urogenital tract. The underlying mechanisms and the functionality of surface aggregation factors are not fully understood; on the one hand aggregation ability may not be the only components responsible for adhesion, and some of the criteria may be part of a complex mechanism that enables the microorganisms to interact with the host and to exert their beneficial effects (García-Cayuela et al., 2014). On the other hand, important mechanisms involved in this process are thought to include adherence as well as colonization of the GIT (Nazzaro et al., 2012; Skrzypczak et al., 2015). The expression of adhesins on the cell surface could induce cell aggregation visible as auto-aggregation. Aggregation promoting factors of lactobacilli differ in size, from 2 kDa in the strain *Lactobacillus gasseri* 2459–318.6 kDa in *L. paracasei* subsp. *paracasei* BGNJ1-64 (Boris et al., 1997; Miljkovic et al., 2015). Interestingly we have reported a new group of aggregation promoting factors of a high molecular mass, recently discovered in LAB (Kojic et al., 2011; Miljkovic et al., 2015). They differ in size and primary structure; however, they share similar structural organization and functions because they are composed of a large number of collagen-binding and CnaB-like domains (Miljkovic et al., 2015). Currently, no experimental evidence exists concerning the role of these domains in aggregation except for predictions that are based on a *S. aureus* collagen-binding Cna protein that mediates bacterial adherence to collagen. The major differences between the aggregation factors of the LAB and the Cna protein of *S. aureus* are that the primary structure of Cna has a non-repetitive collagen binding A region, followed by a repetitive B region (one–four 23 kDa repeating units B1–B4, depending on the strain). It has been suggested that the A region is involved in collagen binding, while the B region acts as a “stalk” that projects the A region from the bacterial surface, facilitating its adherence to collagen (Deivanayagam et al., 2000).

As mentioned above, the AggLb protein is the largest (318.6 kDa) aggregation factor of lactobacilli responsible for auto-aggregation, collagen binding and pathogen exclusion *in vitro*. AggLb consists of six diverse collagen binding domains (from 13202–15256 Da repeating units) and 20 almost identical CnaB-like domains (a 9916 Da repeating unit). The aim of this study was to investigate the roles of the different domains of the AggLb protein involved in probiotic function; this information might prove useful for its potential application. A series of variants of *aggLb* gene/protein were constructed, and their capability to induce auto-aggregation, binding to collagen and fibronectin, and biofilm formation was analyzed. It was concluded that AggLb could provide all of these functions: aggregation and binding to collagen and fibronectin as well as biofilm formation. Interestingly, strong auto-aggregation, collagen and fibronectin binding capacities of AggLb are negatively correlated with the ability of biofilm formation.

## Materials and Methods

### Bacterial Strains, Plasmids, and Growth Conditions

The strains, their derivatives and plasmids used in this study are listed in **Table 1**. *L. paracasei* was grown in De Man-Rogosa-Sharpe (MRS; Merck GmbH, Darmstadt, Germany) medium at 30°C. *Lactococcus lactis* subsp. *lactis* was grown at 30°C in M17 medium (Merck) supplemented with 0.5% glucose (GM17). *Pseudomonas aeruginosa* PAO1 and *E. coli* DH5α and M15 used for cloning and propagation of constructs were routinely grown in Luria-Bertani medium (LB) at 37°C with aeration. To obtain solid medium, agar (15 g/l; Torlak, Belgrade, Serbia) was added. Erythromycin was added to a final concentration of 10 μg/ml and 300 μg/ml for LAB and *E. coli*, respectively. Ampicillin and kanamycin were added to a final concentration of 100 μg/ml for *E. coli*. When necessary, 5-bromo-4-chloro-3-indolyl-β-D-galactoside (X-Gal; Fermentas, Vilnius, Lithuania) was added to LB medium plates at a final concentration of 40 μg/ml for blue/white color selection of colonies.

**Table 1:** Bacterial strains and plasmids used in the study.

Strain	General characteristics	Source or reference
*Lactobacillus paracasei* subsp. *paracasei*		
BGNJ1-64	Natural isolate; Agg^+^	Miljkovic et al., 2015
BGNJ1-641	Derivative BGNJ1-64; Agg^-^	Miljkovic et al., 2015
*Lactococcus lactis* subsp. *Lactis*		
BGKP1	Natural isolate; Agg^+^	Kojic et al., 2011
BGKP1-20	Derivative BGKP1; Agg^-^	Kojic et al., 2011
BGKP1-20/pAZIL-pPIAggLb	Derivative BGKP1-20 carrying pPIAggLb	This study
BGKP1-20/pPI4E	Derivative BGKP1-20 carrying pPI4E	This study
BGKP1-20/pPI3C	Derivative BGKP1-20 carrying pPI3C	This study
BGKP1-20/pPI3D	Derivative BGKP1-20 carrying pPI3D	This study
BGKP1-20/pPI3E	Derivative BGKP1-20 carrying pPI3E	This study
BGKP1-20/pPI2B	Derivative BGKP1-20 carrying pPI2B	This study
BGKP1-20/pPI2D	Derivative BGKP1-20 carrying pPI2D	This study
BGKP1-20/pPI2E	Derivative BGKP1-20 carrying pPI2E	This study
BGKP1-20/pPI1A	Derivative BGKP1-20 carrying pPI1A	This study
BGKP1-20/pPI1D	Derivative BGKP1-20 carrying pPI1D	This study
BGKP1-20/pPI1E	Derivative BGKP1-20 carrying pPI1E	This study
BGKP1-20/pKP-Lb	Derivative BGKP1-20 carrying pKP-Lb	This study
*Lc. lactis* subsp. *cremoris*		
MG7284	Prt^-^, Lac^-^, Bac^r^, Fus^r^, Spc^r^	Gasson, 1983
*Escherichia coli*		
DH5α	*supE44 ΔlacU169* (*ø80 lacZΔM15*) *hsdR17 recA1 endA1 gyrA96 thi-1 relA1*	Hanahan, 1983
M15	Nal^s^, Str^s^, Rif^s^, Thi^-^, Lac^-^, Ara^+^, Gal^+^, Mtl^-^, F^-^, RecA^+^, Uvr^+^, Lon^+^	Qiagen
*Pseudomonas aeruginosa*		
PAO1		Laboratory collection
**Plasmids and constructs**		
pGEM-T Easy Vector	3015 bp, Amp^r^, bacterial, non-viral, transient, constitutive, high expression level, cloning vector	Promega
pBScript vector	2958 bp, Amp^r^, cloning vector	Agilent technologies
pCR2.1-TOPO	3908 bp, Amp^r^, Kan^r^, cloning vector	Thermo Scientific
pCRII	3971 bp, Amp^r^, Kan^r^, cloning vector	Thermo Scientific
pQE30	Amp^r^, ColE1 replicon, HIS6 expression vector	Qiagen
pAZIL	Em^r^, shuttle cloning vector	LMBP 9596
pALb35	pAZILSJ derivative carrying 11377 bp SacI fragment of pNJ1 plasmid from BGNJ1-64	Miljkovic et al., 2015
pAggLbXS	*Xba*I-*Sal*I fragment from pALb35 cloned in pAZIL vector	This study
pBS-XP	First part of *aggLb* cloned as *Xba*I-*Pst*I into pBluescript vector	This study
pCR-XP	First part of *aggLb* cloned as *Xba*I-*Pst*I into pCR2.1-TOPO vector	This study
pBS-PS	Second part of *aggLb* cloned as *Pst*I-*Sal*I into pBluescript vector	This study
pBS-XP-1	pBS-SP were partially digested with *Hin*dIII restriction enzyme and ligated (without 1461, 820, and 821 bp)	This study
pBS-XP-4	The same as pBS-XP	This study
pCR-XP-2	pCR-XP were partially digested with *Ssp*I restriction enzyme and ligated (without 630 and 1611 bp)	This study
pCR-XP-3	pCR-XP were partially digested with *Ssp*I restriction enzyme and ligated (without 1611 bp)	This study
pBS-PS-A	The same as pBS-PS (aforementioned)	This study
pBS-PS-B	pBS-PS were partially digested with *Hin*dIII restriction enzyme and ligated (without both fragments of 1410 bp)	This study
pBS-PS-C	pBS-PS were partially digested with *Hin*dIII restriction enzyme and ligated (without 846 and both fragments of 1410 bp)	This study
pBS-PS-D	pBS-PS were partially digested with *Hin*dIII restriction enzyme and ligated (without both fragments of 1410 and 1266 bp)	This study
pBS-PS-E	pBS-PS were partially digested with *Hin*dIII restriction enzyme and ligated (without 846, both fragments of 1410 and 1266 bp)	This study
pBS-PI4E	*Xba*I/*Pst*I fragment from pBS-XP-4 pooled with *Pst*I-*Sal*I fragment from pBS-PS-E, used pBScript vector	This study
pBS-PI3C	*Xba*I/*Pst*I fragment from pCR-XP-3 pooled with *Pst*I-*Sal*I fragment from pBS-PS-C, used pBScript vector	This study
pBS-PI3D	*Xba*I/*Pst*I fragment from pCR-XP-3 pooled with *Pst*I-*Sal*I fragment from pBS-PS-D, used pBScript vector	This study
pBS-PI3E	*Xba*I/*Pst*I fragment from pCR-XP-3 pooled with *Pst*I-*Sal*I fragment from pBS-PS-E, used pBScript vector	This study
pBS-PI2B	*Xba*I/*Pst*I fragment from pCR-XP-2 pooled with *Pst*I-*Sal*I fragment from pBS-PS-B, used pBScript vector	This study
pBS-PI2D	*Xba*I/*Pst*I fragment from pCR-XP-2 pooled with *Pst*I-*Sal*I fragment from pBS-PS-D, used pBScript vector	This study
pBS-PI2E	*Xba*I/*Pst*I fragment from pCR-XP-2 pooled with *Pst*I-*Sal*I fragment from pBS-PS-E, used pBScript vector	This study
pBS-PI1A	*Xba*I/*Pst*I fragment from pBS-XP-1 pooled with *Pst*I-*Sal*I fragment from pBS-PS-A, used pBScript vector	This study
pBS-PI1D	*Xba*I/*Pst*I fragment from pBS-XP-1 pooled with *Pst*I-*Sal*I fragment from pBS-PS-D, used pBScript vector	This study
pBS-PI1E	*Xba*I/*Pst*I fragment from pBS-XP-1 pooled with *Pst*I-*Sal*I fragment from pBS-PS-E, used pBScript vector	This study
pAZIL-pSE	Lactococcal promoter P*lsbB* was cloned into pAZIL vector together with leader sequence of *aggLb* gene as *Sac*I-*Eag*I fragment	This study
pPIAggLb	*Eag*I-*Sal*I fragment cloned from pALb35 into pAZIL-pSE construct	This study
pPI4E	*Eag*I-*Sal*I fragment cloned from pBS-PI4E into pAZIL-pSE construct	This study
pPI3C	*Eag*I-*Sal*I fragment cloned from pBS-PI3C into pAZIL-pSE construct	This study
pPI3D	*Eag*I-*Sal*I fragment cloned from pBS-PI3D into pAZIL-pSE construct	This study
pPI3E	*Eag*I-*Sal*I fragment cloned from pBS-PI3E into pAZIL-pSE construct	This study
pPI2B	*Eag*I-*Sal*I fragment cloned from pBS-PI2B into pAZIL-pSE construct	This study
pPI2D	*Eag*I-*Sal*I fragment cloned from pBS-PI2D into pAZIL-pSE construct	This study
pPI2E	*Eag*I-*Sal*I fragment cloned from pBS-PI2E into pAZIL-pSE construct	This study
pPI1A	*Eag*I-*Sal*I fragment cloned from pBS-PI1A into pAZIL-pSE construct	This study
pPI1D	*Eag*I-*Sal*I fragment cloned from pBS-PI1D into pAZIL-pSE construct	This study
pPI1E	*Eag*I-*Sal*I fragment cloned from pBS-PI1E into pAZIL-pSE construct	This study
pCRII-KPI	First part of KPPvScI cloned as PCR fragment into pCRII vector	This study
pKP-Lb	Hybrid clone; consisting of first part of *aggL* gene as *Pvu*I-*Pst*I fragment and second part of *aggLb* gene as *Pst*I-*Sal*I fragment into pAZIL vector	This study
pQE_30_-AggBS	Fusion His-tagged part of AggLb protein into pQE_30_ expression vector; in order to production of polyclonal antibody	This study


### DNA Manipulations

Electrocompetent *Lc. lactis* subsp. *lactis* BGKP1-20 cells was prepared as described by Holo and Nes (1989). Transformations were done by electroporation using an Eppendorf Electroporator (Eppendorf, Hamburg, Germany), except *E. coli* DH5α and M15, which was transformed by heat shock (Hanahan, 1983). Appropriate agar plates with antibiotics were used for the selection of transformants.

Plasmid DNA from *E. coli* DH5α was isolated by QIAprep Spin Miniprep kit (Qiagen GmBH, Hilden, Germany). Digestion with restriction enzymes was conducted according to the supplier’s instructions (Fermentas). DNA fragments were purified from agarose gels using a QIAquick Gel extraction kit as described by the manufacturer (Qiagen). DNA was ligated with T4 DNA ligase (Agilent technologies, USA) according to the manufacturer’s recommendations.

Specific primers used in this study are listed in section: Construction of the *aggLb* gene variants. KapaTaq DNA polymerase (Kapa Biosystems, Inc., Boston, MA, USA) was used to amplify DNA fragments by PCR using a GeneAmp PCR system 2700 thermal cycler (Applied Biosystems, Foster City, CA, USA). PCR products were purified with a QiaQuick PCR purification kit (Qiagen) according to the protocol of the supplier and sequenced by the Macrogen Sequencing Service (Macrogen, Netherlands). The DNA Strider program was used for open reading frame (ORF) prediction. Commercial pGEM-T-Easy (Promega, Madison, WI, USA), pCR2.1-TOPO (Thermo Scientific) and pCRII (Thermo Scientific) vectors were used for cloning of PCR products.

### Construction of the aggLb Gene Variants

**FIGURE 1:**
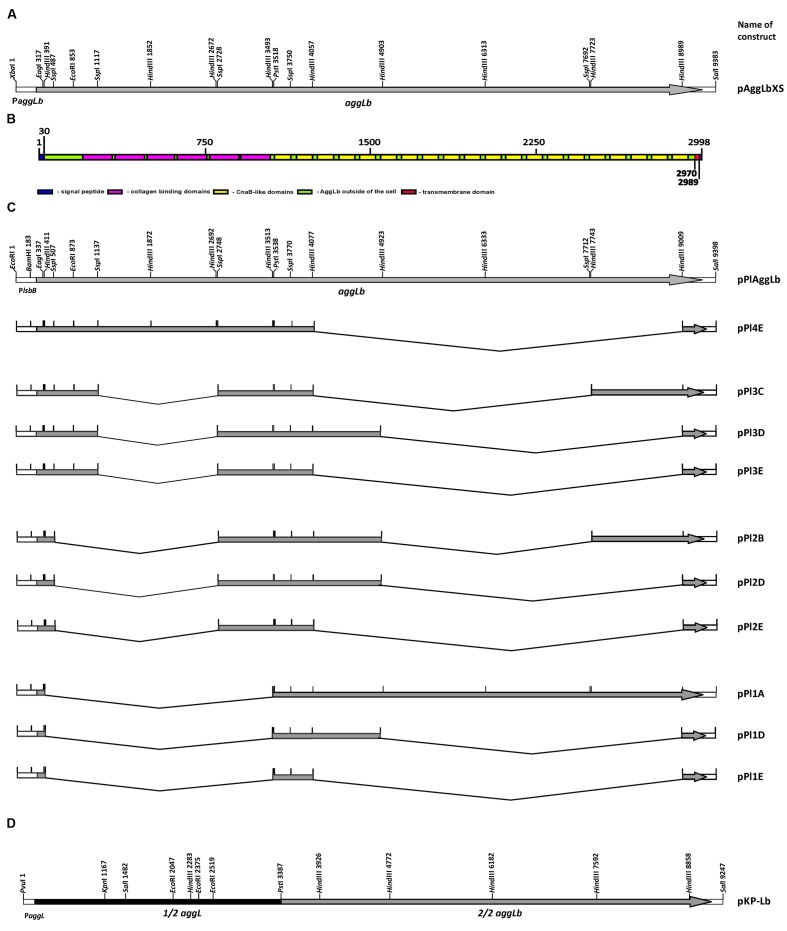
**Schematic representation of strategy for construction variants of *aggLb* gene and hybrid construct.** **(A)** *aggLb*; **(B)** AggLb domain organization (boxes indicate domains of protein); **(C)** series of variants expressed using the lactococcal promoter P*lsbB*; **(D)** hybrid clone pKP-Lb.

From construct pALb35 (Miljkovic et al., 2015) using *Xba*I-*Sal*I restriction enzymes we made shorter construct pAggLbXS carrying only *aggLb* gene, in pAZIL vector (**Supplementary Figure 1A**). *Pst*I restriction site is located in *aggLb* gene at position to divide it into two regions: first containing leader peptide sequence and six collagen binding domains and second containing 20 CnaB-like domains and anchor domain (**Figure 1**). In order to facilitate the construction of a large number of variants, *aggLb* gene was subcloned from pAggLbXS in two parts into pBScript vector (Agilent technologies): first part as *Xba*I-*Pst*I (construct pBS-XP) and second as *Pst*I-*Sal*I fragments (construct pBS-PS; **Supplementary Figure 1A**). Bioinformatic analysis showed that *Hin*dIII (in both fragments; **Supplementary Figures 1B,D**) and *Ssp*I (only in *Xba*I-*Pst*I fragment; **Supplementary Figure 1C**) restriction enzymes dividing AggLb protein to distinct portions that contain the exact number of codons without free base except one in *Xba*I-*Pst*I fragment, so that they can be deleted or combined because they provide in frame junction. Constructs pBS-XP [consisting of three *Hin*dIII fragments of 820 bp, 821 bp (this two cannot be deleted separately since deletion of each fragment changed frame and introduce frameshift mutation) and 1461 bp] and pBS-PS (consisting of four *Hin*dIII fragments of 846 bp, 1266 bp, and two of 1410 bp) were partially digested with *Hin*dIII restriction enzyme and ligated. We successfully constructed pBS-XP-1, pBS-XP-4, pBS-PS-A, pBS-PS-B, pBS-PS-C, pBS-PS-D, and pBS-PS-E (for details see **Table 1** and **Supplementary Figure 1**). From construct pBS-XP fragment carrying *Xba*I/*Pst*I was recloned into pCR2.1-TOPO (since does not contain *Ssp*I restriction site; Thermo Scientific, Lithuania) giving construct pCR-XP, which was additionally partially digested with *Ssp*I restriction enzyme and ligated (constructs pCR-XP-2 and pCR-XP-3; **Supplementary Figure 1**). In next step, different constructs containing deletion in first part (pBS-XP-1, pBS-XP-4, pCR-XP-2, and pCR-XP-3) were combined with constructs containing deletion in second part (pBS-PS-A, pBS-PS-B, pBS-PS-C, pBS-PS-D, and pBS-PS-E) in pBScript vector (for details see **Table 1** and **Figure 1**). In order to obtained expression in lactococci, lactococcal promoter P*lsbB* (Uzelac et al., 2015) was cloned into pAZIL vector together with leader sequence of *aggLb* gene as *Sac*I-*Eag*I fragment (construct pAZIL-pSE). After that different combinations of variants from pBScript vector were cloned as *Eag*I-*Sal*I fragments into pAZIL-pSE (for details see **Table 1** and **Figure 1**). *Lc. lactis* subsp. *lactis* BGKP1-20 was transformed with chosen constructs and expression of different AggLb variants were confirmed by Dot blot analysis using anti-AggLb antibody.

In addition, using template clone KPPvScI (Kojic et al., 2011) and specific set of primers: KPFw (5′GCAAAGCGCCATTCGCC3′), KPPstIRev (5′CGTTCCTTCTGCAGTTCCAC3′), after PCR amplification, we obtained clone pCRII-KPI. *Bam*HI-*Pst*I fragment containing first part of AggL (aggregation factor from *Lc. lactis* subsp. *lactis* BGKP1) was recloned from pCRII-KPI into pBS-PS, from which entire hybrid molecule as *Bam*HI/*Xho*I was transferred to pAZIL vector (digested with *Bam*HI/*Sal*I) and finally obtained clone was named as pKP-Lb (**Table 1**).

### Auto-aggregation Assay

The first step of screening strains was visual auto-aggregation assay. The aggregation phenotype was scored as positive when clearly visible snowflakes-like particles, formed by aggregated cells, gravitated to the bottom of the tube, forming a precipitate and leaving clear supernatant.

The auto-aggregation ability of the selected strains and derivatives was tested according to García-Cayuela et al. (2014) with minor modifications. Briefly, cells of overnight culture were harvested by centrifugation (5000 × *g*, 10 min, 4°C), washed twice with phosphate-buffered saline – PBS (10 mM Na_2_HPO_4_, 1 mM KH_2_PO_4_, 140 mM NaCl, 3 mM KCl, pH 7.1) and resuspended in the same buffer. The mixture was vortexed and incubated at 30°C for a period of 5 h. Absorbance (OD_600_) was measured at different time points. Percentage of auto-aggregation was determined using the equation: [1 - (A_t_/A_0_) × 100] where A_t_ represents the absorbance at different time points (1, 2, 3, 4 and 5 h) and A_0_ is absorbance at time 0. Auto-aggregation assay was done in three independent experiments. Data are presented as average of absorbance values from three independent experiments per each strain. The significance was determined by Student’s *t*-test.

### Biofilm Formation Assay

The ability of selected strains and derivatives to form biofilm was assayed in microtiter plates as previously described by Peter et al. (2013). *P. aeruginosa* PAO1 and *E. coli* DH5α were used as positive and negative control strains, respectively. Additionally, PBS buffer was included to ensure that the influence on biofilm formation by strains (resuspended in the same buffer) not attributed to a non-specific binding effect to crystal violet. The results are presented as average of absorbance values from three independent experiments per each strain. The significance was determined by Student’s *t*-test.

### Collagen and Fibronectin Binding Assays

The wells of Maxisorb plates (Nunc, Roskilde, Denmark) were coated with type I collagen (from rat tail, BD Bioscience, Franklin Lakes, NJ, United States; 100 μg/ml) and human fibronectin (Serva, Heidelberg, Germany; 100 μg/ml) for 16 h at 4°C. The collagen binding ability of the selected strains and derivatives was tested according to Miljkovic et al. (2015), while the ability of tested strains and derivatives to bind to fibronectin was assayed as previously described by Ahmed et al. (2001). After immobilization, wells were washed with PBS and blocked with 2% BSA in PBS. Upon removal of BSA solution and washing wells with PBS, the test cultures (100 μl, 10^8^ CFU/ml) were added and plates were incubated on an orbital platform shaker for 2 h at 37°C. Non-adherent cells were removed by washing the wells three times with 200 μl of PBS. The adhered cells were fixed at 60°C for 20 min and stained with crystal violet (100 μl/well, 0.1% solution) for 45 min. Wells were subsequently washed tree times with PBS to remove the excess stain. The stain bound to the cells was dissolved by 100 μl of citrate buffer (pH 4.3). The absorbance was measured at 570 nm, after 45 min, using the microtiter plate reader. Collagen and fibronectin binding was assayed as described above and the average of absorbance values from three independent experiments per each strain was presented. The significance was determined by Student’s *t*-test.

### Determination of Relationships between Auto-Aggregation, Collagen/Fibronectin Binding, and Biofilm Ability of Transformants Carrying Different Variants of the *aggLb* Gene

Plots of correlation were produced using Python 2.7.8 and scipy library (version 0.14.0).

### Production of Polyclonal Antibody

Since whole AggLb protein was not able to be expressed in *E. coli* the part of AggLb protein containing the inter region of 190 amino acids between collagen binding and CnaB-like domains (from 1096 aa to 1286 aa) present in all variants was expressed using pQE_30_ vector with 6 × His tag (Qiagen) for production of anti-AggLb polyclonal antibody. Using clone pALb35 (Miljkovic et al., 2015), *Hin*dIII fragment of 560 bp containing *Pst*I restriction site was cloned into pBScript. This fragment was recloned from pBScript vector as *Bam*HI/*Sal*I in frame into expression vector pQE_30_ with 6 × His tag (pQE_30_-AggBS). Fusion His-tagged protein was expressed in *E. coli* M15 cells. His-tag affinity purification of part of AggLb protein was conducted under denaturing conditions: the refolding method using urea to disrupt non-covalent bonds and increase protein solubility was used to solubilise and make the His-tagged AggLb more accessible to the nickel-nitrilotriacetic acid (Ni-NTA) resin. Purification of the fusion protein was applied according to protocol recommended by The QIAexpressionist. The eluted protein was dialyzed by ultrafiltration (Centrifugal Filter Units, Amicon Ultra-15 Centrifugal Filter Devices, 3K, Millipore). Polyclonal antibodies were produced by immunization of mice with the synthetic or purified fusion proteins in animal house of ICGEB, Trieste, Italy.

### Dot Blotting

Samples (2 μl of serial dilutions of total proteins dissolved in buffer which contains: 100 mM NaH_2_PO_4_, 10 mM Tris-HCl, 8 M urea, pH 8.0) were loaded into a PVDF membrane (Merck Millipore, Darmstadt, Germany) by directly spotted on membrane as described by Niedergang et al. (2000). The same quantity of non-diluted samples was loaded on PAGE-SDS gel stained with Coomassie brilliant blue (**Supplementary Figure 2**). Membrane was incubated with 10% skim milk diluted in Tris-buffered saline containing 0.1% Tween 20 (TBS-T) over night at 4°C in order to block non-specific reactions. Following blocking, the membrane was incubated 1 h at room temperature with gentle agitation in dilutions of primary antibody (mouse polyclonal antibody anti-AggLb-Ab). Primary antibodies were diluted in 5% skim milk diluted in TBS-T. After washing three times in TBS-T for 15 min, membrane was incubated for 1 h with horseradish peroxidase-labeled anti-mouse IgG (A9044 anti-mouse; Sigma, Germany) at a 1:10000 dilution in 5% skim milk diluted in TBS-T. The blots were washed three times in TBS-T for 15 min. Spots were detected using EMD Millipore Immobilon^TM^ Western Chemiluminescent HRP Substrate (ECL; Fisher Scientific, USA) following the manufacturer’s instructions.

**FIGURE 2:**
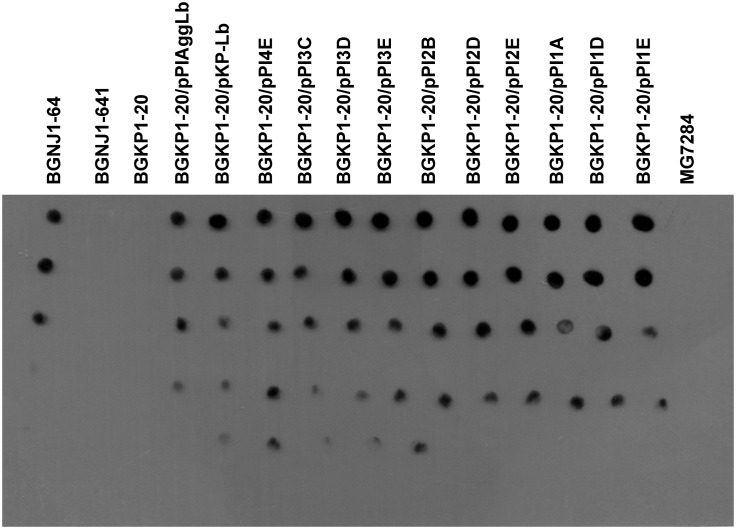
**Dot blot using anti AggLb antibody.** Total proteins of the wild-type strain and of derivatives harboring the different *aggLb* variants in *Lc. lactis* subsp. *lactis* BGKP1-20 strain.

## Results

### Construction of the AggLb Variants

We performed functional studies of the various domains of the AggLb protein. To produce many different domain variants of the AggLb protein, the *aggLb* gene was subcloned into two parts *Sac*I-*Pst*I and *Pst*I-*Sal*I fragments, using the pBscript vector. Both cloned fragments first partially digested using the *Hin*dIII restriction enzyme, and the first part of the gene was also digested using *Ssp*I; importantly, both of these enzymes leave the residual *aggLb* gene in frame. After obtaining different variants of both fragments they were combined to obtain constructs with different numbers of collagen binding and CnaB-like domains. The construct pPI1E did not contain any collagen binding domains and contained only two CnaB-like domains, whereas pPIAggLb contained the complete *aggLb* gene. For details of all the constructs see **Figure 1** and **Table 1**. All the different combinations were recloned into the pAZIL vector using the lactococcal promoter P*lsbB* to provide identical transcription activity of all the constructs (Uzelac et al., 2015). The constructs (**Figure 1**; **Table 1**) were transformed into *Lc. lactis* subsp. *lactis* BGKP1-20 (the lactococcal derivative BGKP1-20 was used because the original lactobacilli strains had an extremely low efficiency of transformation) and expression was analyzed by Dot blot (**Figure 2**) using an anti-AggLb antibody raised against the transitional region covering the last part of the first region and the beginning of the second subclone of AggLb because this part is present in all of the constructs. Similar expression was obtained for all of the constructs regardless of the length of the protein (34.2 kDa pPI1E, 63.9 kDa pPI1D, 65.0 kDa pPI2E, 87.6 kDa pPI3E, 94.8 kDa pPI2D, 117.3 kDa pPI3D, 132.0 kDa pPI3C, 139.3 kDa pPI2B, 145.5 kDa pPI4E, 207.3 kDa pPI1A, and 318.6 kDa pPIAggLb). In addition, the hybrid molecule pKP-Lb (314.2 kDa), consisting of the first part of the lactococcal *aggL* gene from *Lc. lactis* subsp. *lactis* BGKP1 (Kojic et al., 2011) as a *Pvu*I-*Pst*I fragment and a second part of the lactobacilli *aggLb* gene from *L. paracasei* subsp. *paracasei* BGNJ1-64 as a *Pst*I-*Sal*I fragment, was constructed (**Figure 1D**; **Table 1**). All of the variants constructed were used for functional assays in order to determine the role of various domains of the AggLb aggregation protein. The correct in-frame joining of all the fragments was confirmed by DNA sequencing and expression analysis using a Dot blot (**Figure 2**; **Supplementary Figure 2**).

### Auto-Aggregation Ability of Transformants Carrying Different Variants of the *aggLb* Gene

**FIGURE 3:**
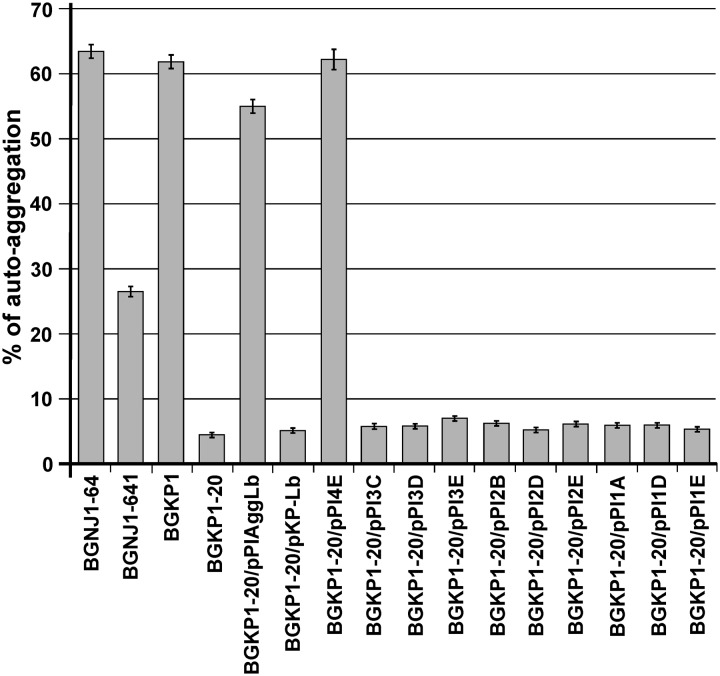
**Comparison of the auto-aggregation ability of the wild-type strain and of derivatives harboring the different *aggLb* variants in *Lc. lactis* subsp.** *lactis* BGKP1-20 strain after 5 h incubation at 30°C. Auto-aggregation ability is expressed as percentages. The error bars represent standard deviations of three independent observations.

**FIGURE 4:**
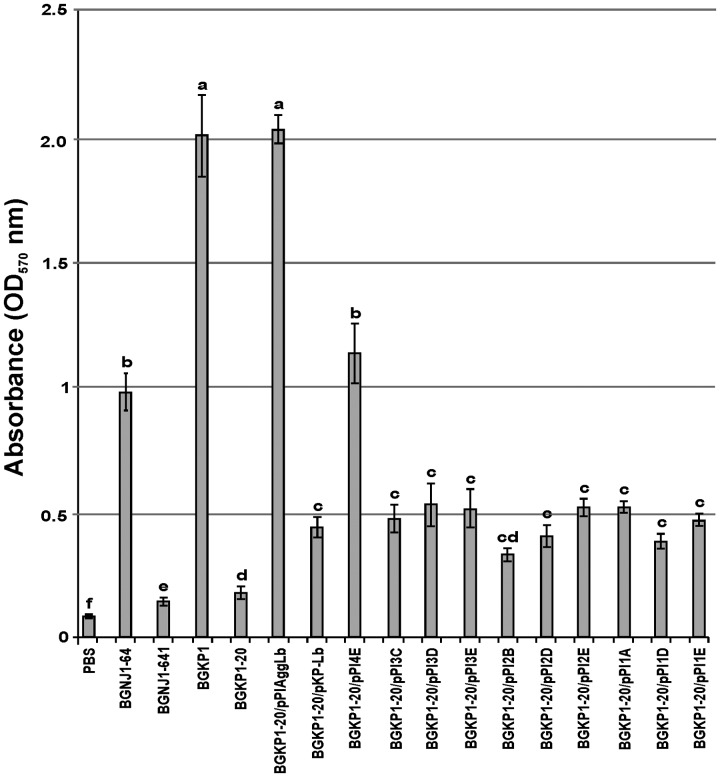
**Graphical presentation of results obtained in collagen-binding assay of selected strains and derivatives to immobilized collagen in microtiter plates.** Results were expressed as average of normalized A_570_ values. The error bars show the standard deviations. In each column, the values with different superscript letters differ significantly (*p* < 0.001).

**FIGURE 5:**
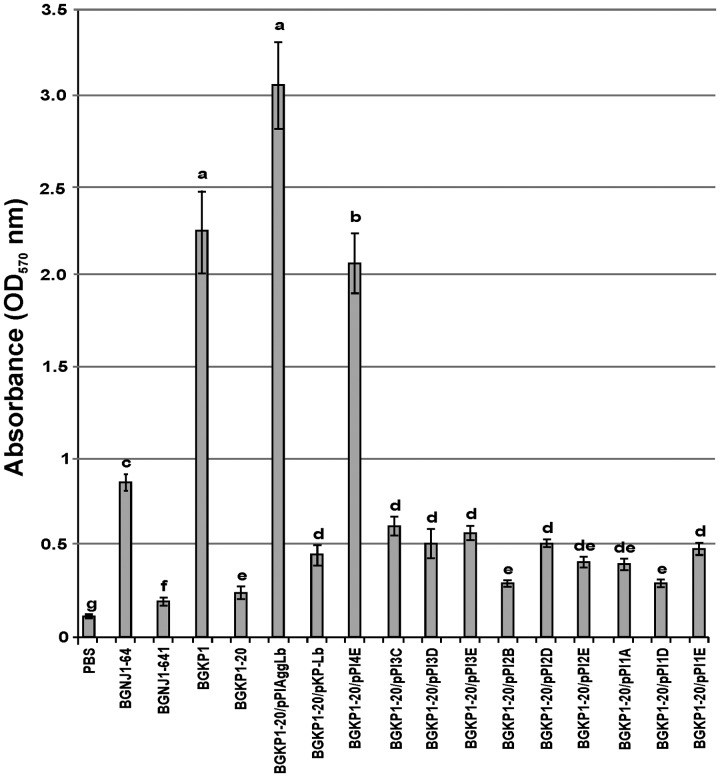
**Graphical presentation of results obtained in fibronectin-binding assay of selected strains and derivatives to immobilized fibronectin in microtiter plates.** Results were expressed as average of normalized A_570_ values. The error bars show the standard deviations. In each column, the values with different superscript letters differ significantly (*p* < 0.001).

The auto-aggregation ability of the wild-type strain and of the derivatives harboring the different variants of *aggLb* in the *Lc. lactis* subsp. *lactis* BGKP1-20 (see above) was measured for a period of 5 h, and the results are presented in **Supplementary Table 1**. We concluded that only the constructs carrying all six collagen binding domains and the first two CnaB-like domains were able to strongly auto-aggregate (BGKP1-20/pPI4E; **Figure 3**; **Supplementary Table 1**). Alternatively, the absence of the other CnaB-like domains, did not cause a significant effect on auto-aggregation (BGKP1-20/pPI3C, BGKP1-20/pPI3D, BGKP1-20/pPI3E, BGKP1-20/pPI2B, BGKP1-20/pPI2D, BGKP1-20/pPI2E, BGKP1-20/pPI1A, BGKP1-20/pPI1D, and BGKP1-20/pPI1E; **Figure 3**; **Supplementary Table 1**). It is also interesting to note that an additive effect dependent on the number of collagen binding domains on auto-aggregation was not linear, indicating that individual collagen binding domains do not have the same contribution. Careful observation revealed that the derivatives BGKP1-20/pPI2E, BGKP1-20/pPI1A, and BGKP1-20/pPI1E formed small aggregates (resembling sand or dust) that did not contribute to the rapid aggregation of the cells. Nevertheless, a negligible level of aggregation that was visible after overnight growth in a test tube was often observed in our collection of LAB. This observation may indicate a relationship between the type and number of collagen binding domains and/or CnaB-like domains within the aggregation factor(s) and the level or types of auto-aggregation. It was, therefore, concluded that the auto-aggregation ability of strains/derivatives was directly dependent on the collagen binding domains, while the 18 C-terminal CnaB-like domains were not required for auto-aggregation. Transformants of *Lc. lactis* subsp. *lactis* BGKP1-20 carrying the hybrid construct pKP-Lb composed of the first part of the *aggL* gene (carrying three collagen binding domains originating from the *Lc. lactis* subsp. *lactis* BGKP1) and the second part of the *aggLb* gene were unable to form big aggregates, which indicated that the resulting hybrid molecule was not functional in strong auto-aggregation, collagen, or fibronectin binding (BGKP1-20/pKP-Lb; **Figures 3–5**) as wild-type strains (*L. paracasei* subsp. *paracasei* BGNJ1-64 and/or *Lc. lactis* subsp. *lactis* BGKP1).

### Collagen and Fibronectin Binding Ability of the Transformants Carrying Different Variants of the *aggLb* Gene

In our previous studies, we found that isolates carrying the *aggL* or *aggLb* genes exhibited a direct correlation between auto-aggregation and their collagen binding ability (Miljkovic et al., 2015). All domain variants of the *aggLb* gene constructed in this study were tested for the ability to bind to collagen and fibronectin. Transformants carrying the different constructs adhered to immobilized collagen (**Figure 4**) and fibronectin (**Figure 5**) to different extents. Significant differences in the adherence to immobilized collagen and fibronectin were apparent between aggregation-positive strains (*L. paracasei* subsp. *paracasei* BGNJ1-64 and *Lc. lactis* subsp. *lactis* BGKP1) and their aggregation-negative derivatives (*L. paracasei* subsp. *paracasei* BGNJ1-641 and *Lc. lactis* subsp. *lactis* BGKP1-20) and also between strains carrying the first part of the *aggLb* gene (consisting of six collagen binding domains and the first two CnaB-like domains; BGKP1-20/pPIAggLb, BGKP1-20/pPI4E) and those variants that had only two or fewer collagen binding domains; these results indicate a role of the collagen binding domains in the interaction with collagen and fibronectin, but the last 18 CnaB-like domains are not indispensable (**Figures 4** and **5**). As observed in other experiments reported in this study (see above), we noticed that the additive effect dependent on the number of collagen binding domains was much lower than the impact of the specific collagen binding domains (II, III, and IV). The specific binding of AggLb to collagen and fibronectin was dependent on the collagen binding domains in a manner similar to the auto-aggregation ability. It appears that all the three phenotypes (auto-aggregation, collagen and fibronectin binding) are determined by the presence of the same structures of the AggLb protein such as the collagen binding domains.

### Biofilm Formation of the Transformants Carrying Different Variants of the *aggLb* Gene

**FIGURE 6:**
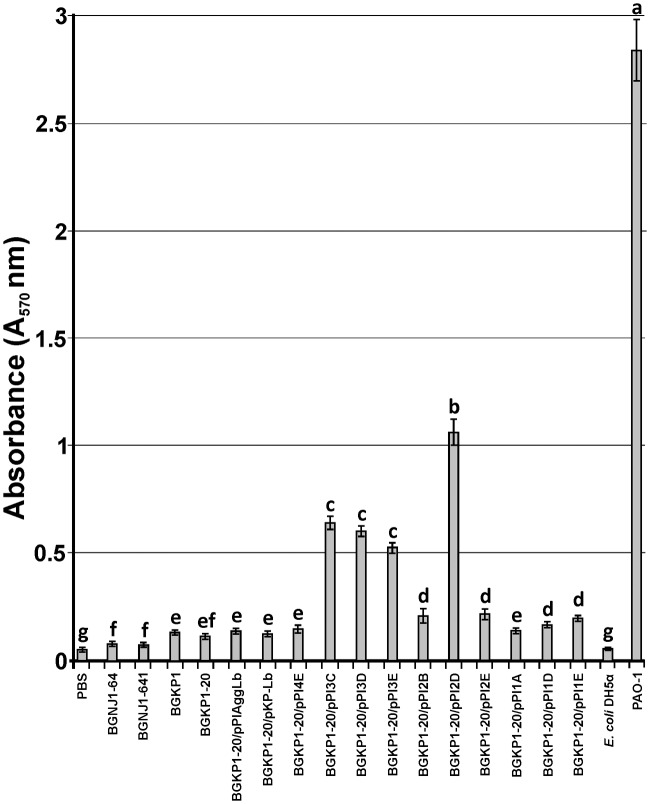
**Graphical presentation of results obtained in biofilm formation assay of selected strains and derivatives (including control strains) to form biofilms in microtiter plates.** Results were expressed as average of normalized A_570_ values. In each column, the values with different superscript letters differ significantly (*p* < 0.05).

We determined the role of the AggLb in biofilm formation. Its ability to form biofilms was tested in the wild-type strain, aggregation deficient derivatives and transformants carrying different variants of *aggLb* using the adherence of the cells to the surfaces of microtiter plates. The strongest biofilm formation was observed for the transformant carrying the construct pPI2D, followed by pPI3C, pPI3D, and finally, pPI3E (**Figure 6**). A comparative analysis of the variants led to the conclusion that the biofilm formation ability has a negative correlation with auto-aggregation, collagen, and fibronectin binding. It appears that the presence of collagen binding domains determines the formation of certain structures on AggLb that play a role in the interaction with collagen and fibronectin, but simultaneously enable the cells to auto-aggregate (pPI4E). Most likely, the absence of the collagen-binding domain (especially II, III, and IV) allows other structures to come to the fore (i.e., they are unmasked) which promotes biofilm formation. The difference between pPI1D and pPI2D is limited to the presence of a sixth collagen binding domain of AggLb in pPI2D (**Figure 1**; **Table 2**); thus, this result indicates that this domain is probably required in combination with the other domain(s) to allow biofilm formation.

**Table 2:** Representation of domain organization series of AggLb variants.

Name of construct	No. of collagen binding domains	No. of CnaB like domains	Molecular mass of expressed protein (kDa)
pPIAggLb	6	20	318.6
pPI4E	6	2	145.5
pPI3C	2 (hybrid of I-V, and VI)	7	132.0
pPI3D	2 (hybrid of I-V, and VI)	5	117.3
pPI3E	2 (hybrid of I-V, and VI)	2	87.6
pPI2B	1 + 1/2 (½ of V and VI)	10	139.3
pPI2D	1 + 1/2 (½ of V and VI)	5	94.8
pPI2E	1 + 1/2 (½ of V and VI)	2	65.0
pPI1A	0	20	207.3
pPI1D	0	5	63.9
pPI1E	0	2	34.2


### Relationships between Collagen/Fibronectin Binding and Biofilm Ability of Transformants Carrying Different Variants of the *aggLb* Gene

We established correlations between auto-aggregation, collagen/fibronectin binding and biofilm formation ability of transformants carrying different variants of the *aggLb* gene. A comparative analysis of the variants led to the conclusion that the biofilm formation ability has a negative correlation with auto-aggregation – R^2^ squared 0.312 (**Supplementary Figure 3A**), binding to collagen – R^2^ squared 0.260 (**Supplementary Figure 3B**), binding to fibronectin – R^2^ squared 0.242 (**Supplementary Figure 3C**). In addition using Python 2.7.8 and scipy library (version 0.14.0) we proved positive correlation between auto-aggregation and collagen binding – R^2^ squared 0.652 (**Supplementary Figure 3D**) and aggregation and fibronectin binding – R^2^ squared 0.636 (**Supplementary Figure 3E**).

## Discussion

The adhesion of lactic acid bacteria to epithelial and mucosal surfaces is thought to be a rather complex process involving many different factors (Buck et al., 2005). The ability of lactobacilli to aggregate has been linked to their role as probiotic factors (García-Cayuela et al., 2014). The data of the literature suggest that the Apf-like proteins may contribute to the survival of *L. acidophilus* during its transit through the digestive tract and, potentially, may participate in the interactions with the host intestinal mucosa (Goh and Klaenhammer, 2010). Considering the importance of aggregation phenomena for human health, the experiments described in this study were mainly focused to determine the contribution of the different domains and repeats of the AggLb protein on the modulation of the aggregation phenotype. Additionally, our results have proven the existence of a direct relationship between strong auto-aggregation, collagen or fibronectin binding and biofilm formation.

Biofilms of lactobacilli can be found in many natural environments (Lebeer et al., 2007). Because the gastrointestinal tract is an important target for probiotics, some factors related to this niche have been investigated in the past decade. It was of interest to study the possible relationship between aggregation ability and biofilm formation. It has been reported that the agglutination protein AggA is required for the aggregation and increased biofilm formation of a hyper-aggregating mutant of *Shewanella oneidensis* MR-1 (De Windt et al., 2006). An insertional mutant of *aggA* resulted in the loss of aggregation properties and ability to form a biofilm. Additionally, the SasC protein of a pathogenic *S. aureus* strain was involved in cell aggregation, biofilm formation and colonization during infection. The N-terminal domain of the SasC protein was involved in the production of large cell aggregates, in the attachment to polystyrene, and in increased biofilm formation (Schroeder et al., 2009). Aggregation and biofilm formation are multicellular processes that allow a community to be more resistant to stress conditions. Given that these are similar processes, it is not surprising that the same protein may be involved in both functions. Since biofilm formation is important in food spoilage and pathogenic bacteria because it results in high resistance to different treatments, it is important to identify and characterize the active components that could inhibit bacterial biofilm formation (Söderling et al., 2011; Furukawa, 2015).

The ability to strongly aggregate and adhere to collagen and fibronectin is inversely correlated with the biofilm formation, (if the ability to strongly aggregate and bind collagen and fibronectin is stronger the ability of biofilm formation is less; **Figures 3–5**; **Supplementary Figure 3**). Therefore, it seems that the lack of collagen binding domains II, III, and IV in the AggLb protein results in the reduced auto-aggregation, collagen and fibronectin binding and increases the propensity of the cells to form a biofilm. A comparative regression analysis of AggLb variants containing a constant number of CnaB-like domains and a different number of collagen binding domains (pPI4E, pPI3E, pPI2E, and pPI1E; pPI3D, pPI2D, and pPI1D; **Figures 4** and **5**) showed a correlation of binding to collagen or fibronectin, and an increase in biofilm formation (**Supplementary Figure 3**).

Our results indicate that the region responsible for the strong auto-aggregation, collagen and fibronectin binding is located on the N-terminus of the AggLb aggregation protein; transformants that carried the construct pPI4E, which contained only the N-terminal part, exhibited a strong aggregation capability, as did as clones that harbored the complete gene. Deletion studies of the AggLb protein showed that all three functions dependent on the collagen binding domains II, III, and IV, and their deletion leads to a complete loss of strong aggregation ability. These three domains are critical for function of AggLb in strong auto-aggregation, binding to collagen and fibronectin, either through direct and specific interaction with proteins of the matrix or by changing the properties of the cell surface. Multiple CnaB-like domains likely function as an antenna which exposes the collagen binding domains to the surface to improve target protein interactions. The CnaB-like domains in AggLb cannot be considered as the domains responsible for the direct interaction with collagen or fibronectin, but they can strengthen the interaction between the collagen binding domains and collagen or fibronectin. Also, we noted that because the first and last CnaB-like domains had sequence heterogeneity compared to the other 18 domains, it is possible they may have a different but not strong effect on AggLb function. We can conclude that the presence of the collagen binding domains predominantly determined the adhesive function of the AggLb protein. In addition, combination of domains from lactobacilli (AggLb) and lactococci (AggL; hybrid molecule – BGKP1–20/pKP-Lb) did not resulted in functional protein in strong auto-aggregation, collagen, or fibronectin binding. The results obtained in this study have demonstrated that a protein may exert different functions depending on physicochemical properties of the bacterial surfaces, and this probably depends on the structure and conformation variants of AggLb. The removal of certain domain(s) not only eliminated certain functions but also resulted in other domain(s) coming to the fore and allowing the protein to assume another function. In our previous publication we have noticed one strain BGGR2-68 that simultaneously exhibits both functions strong auto-aggregation and biofilm formation (Miljkovic et al., 2015). It would be interesting to determine whether these two functions in this strain are associated with one the same protein or independent. This will be the subject of further research.

These results bolster the hypothesis that in the *S. aureus* collagen-binding Cna protein, the collagen binding A region is responsible and sufficient for collagen binding, while the B region aids as a “stalk” that projects the A region from the bacterial surface to facilitate the bacterial adherence to collagen. Such a B region assembly could result in flexibility, stability, and positioning the ligand-binding A region away from the bacterial cell surface (Deivanayagam et al., 2000). The difference between AggLb and the Cna protein is that the aggregation promoting factor contains repetitive collagen binding domains (six very heterogeneous units with less than 26% identity) that have different contributions to strong auto-aggregation, collagen, and fibronectin binding (II, III, and IV showed the most significant effects), as well as to biofilm formation. It is important to note that even if AggLb is composed of two collagen binding domains, it is not able to provide strong auto-aggregation. In contrast in Cna, this is accomplished with a single domain, indicating that it is important which of the domains is/are present.

## Author Contributions

MK conceived, designed, and coordinated this study, interpreted all of results and contributed to the preparation of the figures and wrote this paper. MM designed, performed, analyzed the experiments and wrote this paper. BJ and KN provided experimental assistance and contributed to the preparation of the figures. IB performed one part of experiments of production polyclonal antibody. DF and VV provided technical assistance and contributed to the preparation of this paper. All authors reviewed the results and approved the final version of the manuscript.

## Conflict of Interest Statement

The authors declare that the research was conducted in the absence of any commercial or financial relationships that could be construed as a potential conflict of interest.
