# Supplementary material for: Shortening of the Lactobacillus paracasei subsp. paracasei BGNJ1-64 AggLb Protein Switches Its Activity from Auto-aggregation to Biofilm Formation
Source: Front Microbiol. 2016 Sep 8;7:1422. doi: 10.3389/fmicb.2016.01422 (PMC5014864; doi:10.3389/fmicb.2016.01422)
Supplement: Supplementary file 1 [file Table_1.PDF]

Supplementary Material

Shortening of the *Lactobacillus paracasei* subsp. *paracasei* BGNJ1-64 AggLb protein switches its activity from auto-aggregation to biofilm formation

Marija Miljkovic, Iris Bertani, Djordje Fira, Branko Jovcic, Katarina Novovic, Vittorio Venturi, Milan Kojic*

***Correspondence**: Milan Kojic, mkojic@imgge.bg.ac.rs

Supplementary Tables

Supplementary Table 1. Auto-aggregation ability of selected strains and derivatives determined by spectrophotometry measurements (OD_{600}) for a period of 5 hours.

Strain/derivative	% of auto-aggregation				
	1 h	2 h	3 h	4 h	5 h
BGNJ1-64	43.0±1.36	49.5±1.82	55.3±0.69	59.4±1.74	63.5±0.98
BGNJ1-641	15.9±0.55	19.7±0.79	21.3±1.24	23.1±1.41	26.4±0.67
BGKP1	39.4±1.43	46.8±2.02	51.7±1.78	56.3±2.13	61.8±1.14
BGKP1-20	1.3±0.06	1.8±0.11	2.2±0.08	3.5±0.10	4.2±0.07
BGKP1-20/pPIAggLb	30.5±2.01	37.6±1.34	43.1±2.38	48.6±0.85	54.9±1.22
BGKP1-20/pKP-Lb	1.5±0.10	3.1±0.09	3.6±0.07	4.4±0.13	4.9±0.08
BGKP1-20/pPI4E	33.8±1.14	44.1±2.64	48.3±2.24	55.6±1.89	62.1±2.20
BGKP1-20/pPI3C	1.5±0.13	2.8±0.07	3.5±0.11	4.6±0.17	5.4±0.15
BGKP1-20/pPI3D	1.4±0.05	3.3±0.19	4.1±0.27	5.1±0.04	5.8±0.08
BGKP1-20/pPI3E	1.6±0.09	3.1±0.06	3.5±0.17	4.7±0.08	6.6±0.19
BGKP1-20/pPI2B	1.4±0.05	3.0±0.08	3.5±0.09	5.2±0.24	6.2±0.06

BGKP1-20/pPI2D	1.8±0.07	2.6±0.16	3.6±0.05	4.7±0.16	5.4±0.14
BGKP1-20/pPI2E	1.2±0.12	2.7±0.18	4.0±0.06	5.2±0.05	6.1±0.07
BGKP1-20/pPI1A	1.6±0.06	2.8±0.09	4.1±0.10	5.1±0.24	6.0±0.09
BGKP1-20/pPI1D	1.3±0.08	2.7±0.07	4.4±0.14	5.2±0.09	6.1±0.12
BGKP1-20/pPI1E	0.9±0.07	2.5±0.20	3.6±0.05	4.4±0.15	5.3±0.05
