# Supplementary material for: Shortening of the Lactobacillus paracasei subsp. paracasei BGNJ1-64 AggLb Protein Switches Its Activity from Auto-aggregation to Biofilm Formation
Source: Front Microbiol. 2016 Sep 8;7:1422. doi: 10.3389/fmicb.2016.01422 (PMC5014864; doi:10.3389/fmicb.2016.01422)
Supplement: Supplementary file 2 [file Image_1.PDF]

Supplementary Material

Shortening of the *Lactobacillus paracasei* subsp. *paracasei* BGNJ1-64 AggLb protein switches its activity from auto-aggregation to biofilm formation

Marija Miljkovic, Iris Bertani, Djordje Fira, Branko Jovcic, Katarina Novovic, Vittorio Venturi, Milan Kojic[*]

[*]**Correspondence**: Milan Kojic, mkojic@imgge.bg.ac.rs

Supplementary Figures

Supplementary Figures 1

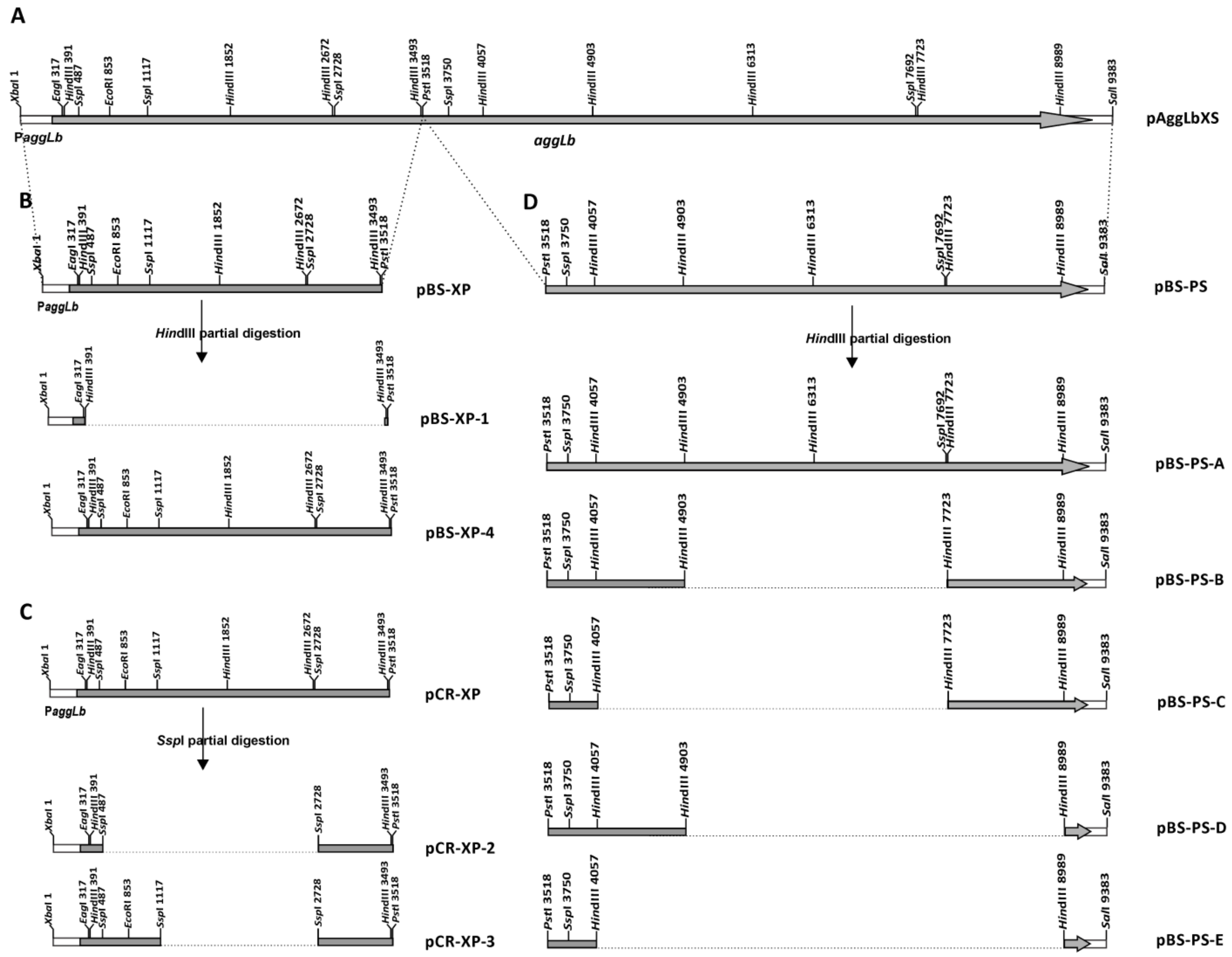

Supplementary Figure 1. Shematic presentation of deletion strategy. (A) *aggLb* gene and surrounding region; (B) Construct of *Xba*I-*Pst*I part of *aggLb* gene in pBluescript vector and its deletion using *Hin*dIII partial digestion; (C) Construct of *Xba*I-*Pst*I part of *aggLb* gene in pCR2.1-TOPO vector and its deletion using *Ssp*I partial digestion; (D) Construct of *Pst*I-*Sal*I part of *aggLb* gene in pBluescript vector and its deletion using *Hin*dIII partial digestion.
