# Supplementary material for: Shortening of the Lactobacillus paracasei subsp. paracasei BGNJ1-64 AggLb Protein Switches Its Activity from Auto-aggregation to Biofilm Formation
Source: Front Microbiol. 2016 Sep 8;7:1422. doi: 10.3389/fmicb.2016.01422 (PMC5014864; doi:10.3389/fmicb.2016.01422)
Supplement: Supplementary file 3 [file Image_2.PDF]

Supplementary Material

Shortening of the *Lactobacillus paracasei* subsp. *paracasei* BGNJ1-64 AggLb protein switches its activity from auto-aggregation to biofilm formation

Marija Miljkovic, Iris Bertani, Djordje Fira, Branko Jovcic, Katarina Novovic, Vittorio Venturi, Milan Kojic*

***Correspondence**: Milan Kojic, mkojic@imgge.bg.ac.rs

Supplementary Figures

Supplementary Figure 2

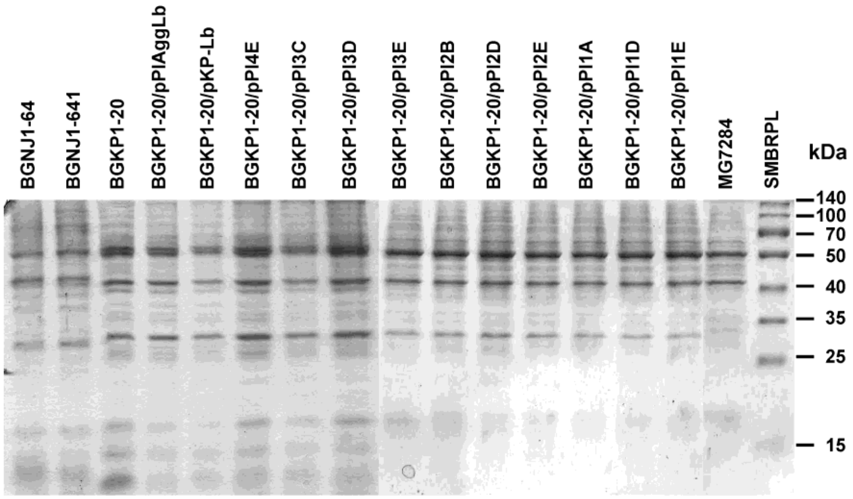

Supplementary Figure 2. Total proteins of the wild type strain and of derivatives harboring the different variants of the *aggLb* gene in the BGKP1-20 strain resolved on 12.5% PAGE-SDS. SMBRPL - Spectra Multicolor Broad Range Protein Ladder (Thermo Fisher Scientific).
