# Supplementary material for: Shortening of the Lactobacillus paracasei subsp. paracasei BGNJ1-64 AggLb Protein Switches Its Activity from Auto-aggregation to Biofilm Formation
Source: Front Microbiol. 2016 Sep 8;7:1422. doi: 10.3389/fmicb.2016.01422 (PMC5014864; doi:10.3389/fmicb.2016.01422)
Supplement: Supplementary file 4 [file Image_3.PDF]

Supplementary Material

Shortening of the *Lactobacillus paracasei* subsp. *paracasei* BGNJ1-64 AggLb protein switches its activity from auto-aggregation to biofilm formation

Marija Miljkovic, Iris Bertani, Djordje Fira, Branko Jovcic, Katarina Novovic, Vittorio Venturi, Milan Kojic*

***Correspondence**: Milan Kojic, mkojic@imgge.bg.ac.rs

Supplementary Figures

Supplementary Figures 3

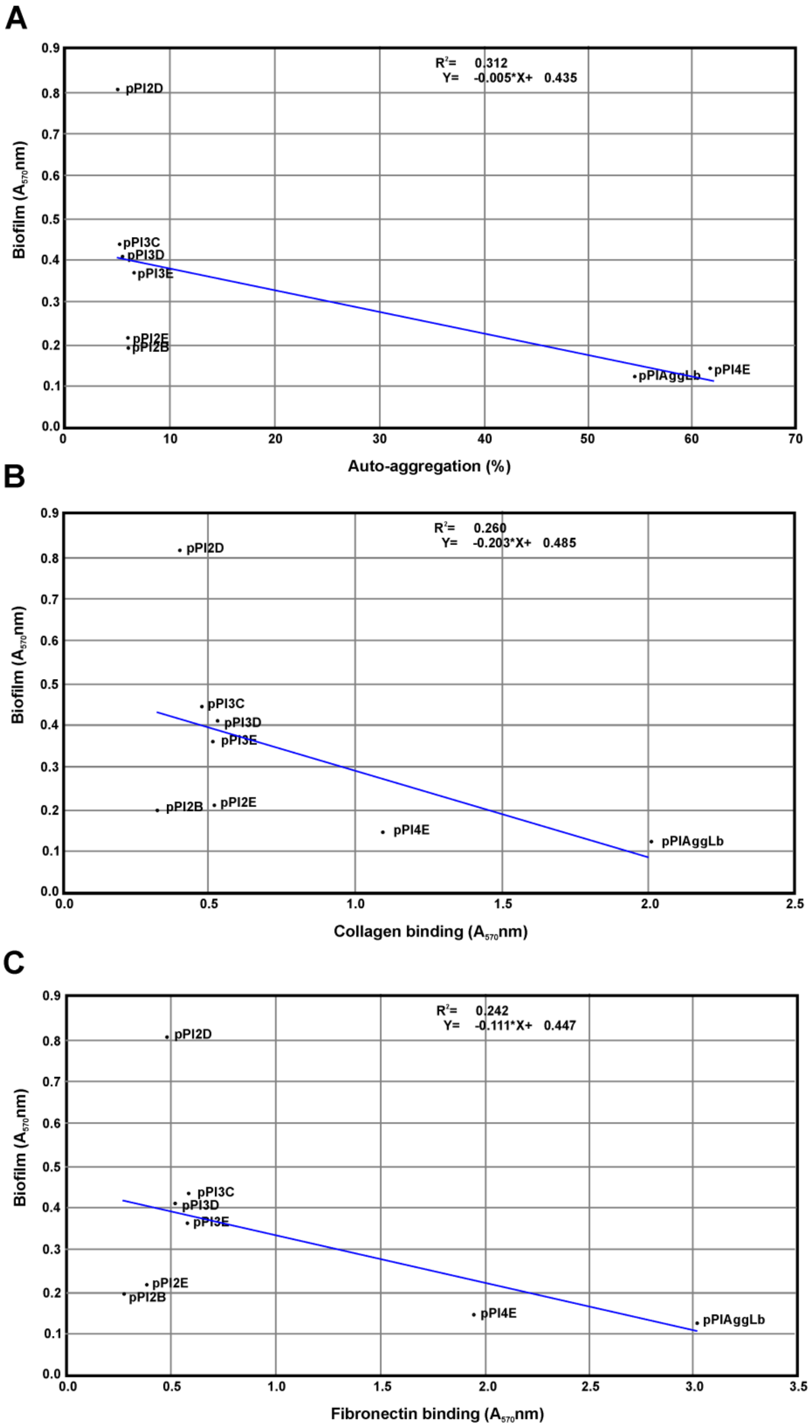
A
R²= 0.312
Y= -0.005*X+ 0.435
pPI2D
pPI3C
pPI3D
pPI3E
pPI2E
pPI2B
pPIAggLb
pPI4E
Biofilm (A570nm)
Auto-aggregation (%)
0.0
0.1
0.2
0.3
0.4
0.5
0.6
0.7
0.8
0.9
0
10
20
30
40
50
60
70
B
R²= 0.260
Y= -0.203*X+ 0.485
pPI2D
pPI3C
pPI3D
pPI3E
pPI2B
pPI2E
pPI4E
pPIAggLb
Biofilm (A570nm)
Collagen binding (A570nm)
0.0
0.5
1.0
1.5
2.0
2.5
C
R²= 0.242
Y= -0.111*X+ 0.447
pPI2D
pPI3C
pPI3D
pPI3E
pPI2E
pPI2B
pPI4E
pPIAggLb
Biofilm (A570nm)
Fibronectin binding (A570nm)
0.0
0.5
1.0
1.5
2.0
2.5
3.0
3.5

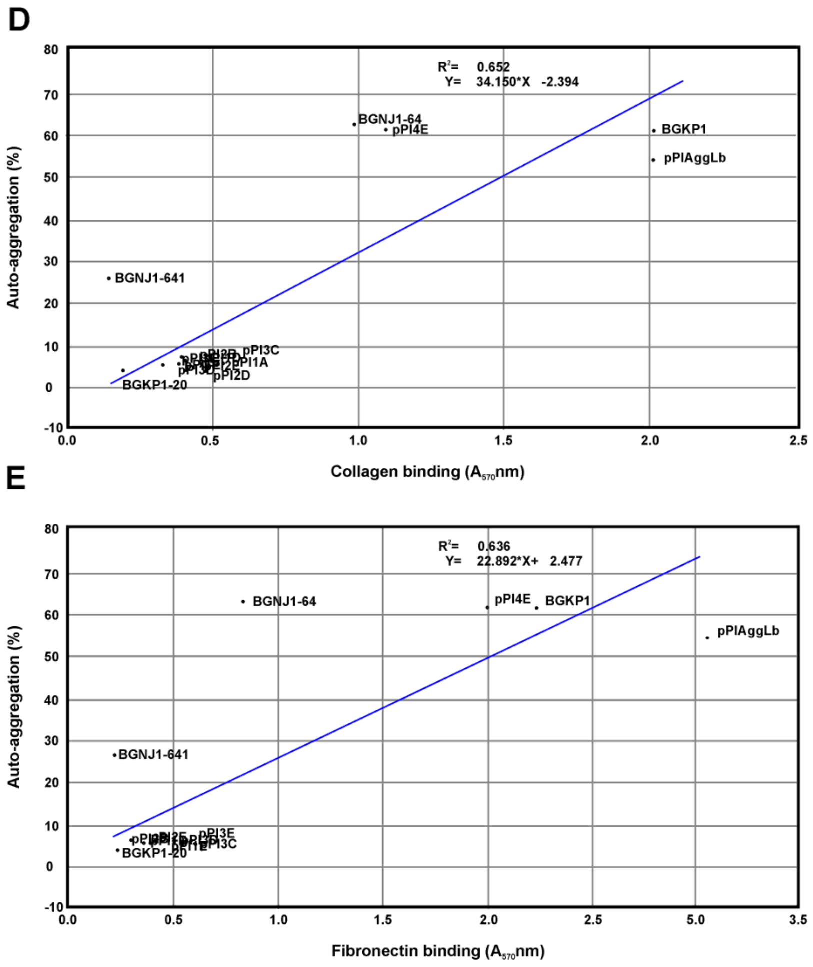

Supplementary Figure 3. Relationships between (A) auto-aggregation and biofilm ability; (B) collagen binding and biofilm ability; (C) fibronectin binding and biofilm ability; (D) auto-aggregation and collagen binding; (E) auto-aggregation and fibronectin binding of transformants carrying different variants of the *aggLb* gene. Plots were produced using Python 2.7.8 and scipy library (version 0.14.0).
